# The clinical features of respiratory infections caused by the *Streptococcus anginosus* group

**DOI:** 10.1186/s12890-015-0128-6

**Published:** 2015-10-26

**Authors:** Shingo Noguchi, Kazuhiro Yatera, Toshinori Kawanami, Kei Yamasaki, Keisuke Naito, Kentaro Akata, Ikuko Shimabukuro, Hiroshi Ishimoto, Chiharu Yoshii, Hiroshi Mukae

**Affiliations:** Department of Respiratory Medicine, Wakamatsu Hospital of the University of Occupational and Environmental Health, Japan, 1-17-1, Hamamachi, Wakamatsuku, Kitakyushu city, 808-0024 Fukuoka Japan; Department of Respiratory Medicine, University of Occupational and Environmental Health, Japan, 1-1, Iseigaoka, Yahatanishiku, Kitakyushu City, 807-8555 Fukuoka Japan

**Keywords:** *S. anginosus* group, Pneumonia, Lung abscess, Bacterial pleurisy, Pleural effusion

## Abstract

**Background:**

The *Streptococcus anginosus* group (SAG) play important roles in respiratory infections. It is ordinarily difficult to distinguish them from contaminations as the causative pathogens of respiratory infections because they are often cultured in respiratory specimens. Therefore, it is important to understand the clinical characteristics and laboratory findings of respiratory infections caused by the SAG members. The aim of this study is to clarify the role of the SAG bacteria in respiratory infections.

**Methods:**

A total of 30 patients who were diagnosed with respiratory infections which were caused by the SAG bacteria between January 2005 and February 2015 were retrospectively evaluated.

**Results:**

Respiratory infections caused by the SAG were mostly seen in male patients with comorbid diseases and were typically complicated with pleural effusion. Pleural effusion was observed in 22 (73.3%) patients. Empyema was observed in half of the 22 patients with pleural effusion. *S. intermedius*, *S. constellatus* and *S. anginosus* were detected in 16 (53.3 %), 11 (36.7 %) and 3 (10.0 %) patients, respectively. Six patients had mixed-infections. The duration from the onset of symptoms to the hospital visit was significantly longer in “lung abscess” patients than in “pneumonia” patients among the 24 patients with single infections, but not among the six patients with mixed-infection. The peripheral white blood cell counts of the “pneumonia” patients were higher than those of the “lung abscess” patients and *S. intermedius* was identified significantly more frequently in patients with pulmonary and pleural infections (pneumonia and lung abscess) than in patients with bacterial pleurisy only. In addition, the patients in whom *S. intermedius* was cultured were significantly older than those in whom *S. constellatus* was cultured.

**Conclusions:**

Respiratory infections caused by the SAG bacteria tended to be observed more frequently in male patients with comorbid diseases and to more frequently involve purulent formation. In addition, *S. intermedius* was mainly identified in elderly patients with having pulmonary infection complicated with pleural effusion, and the aspiration of oral secretions may be a risk factor in the formation of empyema thoracis associated with pneumonia due to *S. intermedius*.

**Electronic supplementary material:**

The online version of this article (doi:10.1186/s12890-015-0128-6) contains supplementary material, which is available to authorized users.

## Background

The *Streptococcus anginosus* group (SAG), which is often referred to as the *Streptococcus milleri* group, has been widely detected in the mouth, the upper respiratory tract, the gastrointestinal tract and the vagina [1]. At least three different species have been classified into the SAG: *S. intermedius*, *S. constellatus* and *S. anginosus* [2, 3].

The important roles of the streptococci in various types of respiratory infections have recently been reported [4–6]. We also reported, based on the results of a clone library analysis of the 16S ribosomal RNA gene, that the streptococci play important roles in the pathogenesis of respiratory infections [7, 8]. Among the various streptococci, the SAG bacteria have been reported to be particularly important in the pathogenesis of respiratory infections [1, 6, 9–12].

It has been reported, because of the unique characteristics of the SAG bacteria, they tend to form abscesses and empyema thoracis [3, 12, 13], and such SAG-based infections account for 13–50 % of all cases of pulmonary abscess and/or empyema thoracis [5, 9, 11, 14]. It is difficult to determine whether they are the causative pathogens of respiratory tract infections when the SAG bacteria are obtained in sputum cultivation because the SAG bacteria are resident members of the flora of both the oral and/or upper respiratory tract. In addition to the culture results, it is therefore important to understand the clinical characteristics and the laboratory findings related to thoracic SAG infection.

We herein retrospectively investigated the clinical characteristics of respiratory infections caused by members of the SAG.

## Methods

### Study design and patients

This retrospective study was conducted at the University of Occupational and Environmental Health, Japan (UOEH) and Wakamatsu Hospital of the UOEH. Patients with positive SAG bacteria cultures from specimens obtained from the respiratory tract, pleural effusion, blood, and other samples from January 2005 to February 2015 in the UOEH, and from April 2011 to February 2015 in Wakamatsu Hospital of UOEH (this hospital was established in April 2011) were enrolled in the present study. Patients were singly enrolled in this study. This study was approved by the Human and Animal Ethics Review Committee of the UOEH (No. 26–229), and was in accordance with the Declaration of Helsinki. The information that was collected included the patients’ age, sex, comorbid diseases, clinical manifestations and the laboratory and radiological findings.

### Diagnostic criteria

Pneumonia was defined by the fulfillment of all three of the following criteria: (1) positive findings for at least one of the assessed clinical symptoms (fever, cough, sputum production, chest pain); (2) the presence of new infiltrates without a cavity or low density areas on chest radiography and/or computed tomography (CT); and (3) positive findings for at least one of the sign(s) of leukocytosis (a white blood cell count (WBC) of ≥ 10,000/μl) and/or increased serum levels of C-reactive protein (CRP). A lung abscess was defined when a cavity or low density area within the infiltrates was confirmed on chest radiography and/or CT in addition to the above clinical criteria. Bacterial pleurisy, which is defined as pleural infection due to bacteria, was macroscopically defined by the existence of purulent pleural fluid or inflammatory cells (predominantly neutrophils) in the pleural effusion in addition to (1) and (3) of the above criteria. Pneumonia and lung abscess, with or without pleural effusion, was described as “pneumonia” and “lung abscess”, respectively, in spite of the associated pleural effusion, while bacterial pleurisy without pneumonia or lung abscess was described as “bacterial pleurisy only”. Complicated parapneumonic effusion was defined by a low glucose level (<40 mg/dL) and high levels of lactate dehydrogenase (>1000 IU/L), or positive gram staining or culture results. Empyema thoracis was defined by the existence of macroscopically observable purulent pleural fluid [15]. Patients who did not fulfill the above-described diagnostic criteria (pneumonia, lung abscess, and bacterial pleurisy only) were excluded from the study.

### Microbiological evaluation

Bacterial cultures were evaluated using a semi-quantitative method [8]. As previously reported, the SAG bacteria were indicated as causative pathogens in the present study by a bacterial volume of “1+ (≥10^3^–10^5^ colony-forming units (CFU)/ml)” in bronchoalveolar lavage fluid (BALF) or “2+ (≥10^6^ CFU/ml)” in sputum samples by reference to the results of quantitative culture, the findings of which included ≥10^6^ CFU/ml in sputum and 10^4^ CFU/ml in BALF [16–18]. The SAG bacteria were also identified as causative pathogens when blood or pleural effusion culture results were found to be positive. As previously described, the isolated bacterial species were identified using a VITEK 2 system (bio-Mérieux) with or without an API 20 STREP system (bio-Mérieux) [19]. Patients who did not fulfill the following criteria were excluded to avoid bacterial contamination to the extent that was possible, even when one of the above diagnostic criteria for pneumonia, lung abscess and bacterial pleurisy was fulfilled: a bacterial volume of “≥1+” in BALF or “≥2+” in sputum samples.

### Statistical analysis

After excluding the 6 patients with mixed infection to clarify the characteristics of SAG respiratory infection, we analyzed the results of 24 patients. All statistical analyses were performed using the SPSS software package (version 19), and a value of *p* < 0.05 was considered to be statistically significant. Continuous variables were compared using the Mann-Whitney (non-parametric) *U*-test, while categorical variables were compared using Fisher’s exact test (2 × 2), as appropriate.

## Results

### Patient characteristics and laboratory findings of 30 patients

**Fig. 1:**
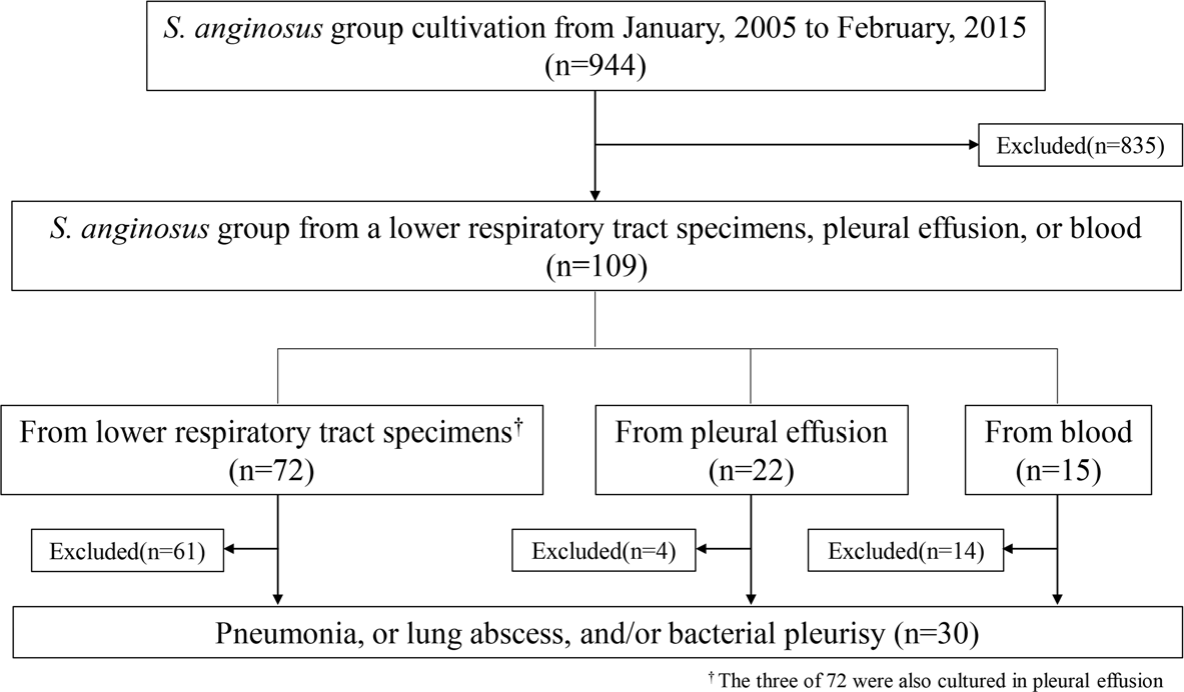
A flow diagram of the patient inclusion and exclusion criteria

Among the 944 patients in whom SAG bacteria were cultured during the study period, 109 patients showed cultures that were positive for SAG bacteria from lower respiratory tract, pleural effusion or blood samples. In these 109 patients, 74 patients were excluded because they did not fulfill the diagnostic criteria of pneumonia, lung abscess or bacterial pleurisy only, and 5 patients were excluded because their bacterial volume, which was calculated semi-quantitatively, did not fulfill the diagnostic criteria. Finally, a total of 30 patients were diagnosed with a respiratory infection (pneumonia, lung abscess, bacterial pleurisy only) due to *S. intermedius*, or *S. constellatus*, or *S. anginosus* (Fig. 1). The clinical characteristics, laboratory findings of the 30 patients are summarized in Table 1. The average age was 68.9 ± 14.2 years, and 22 (73.3 %) of the 30 patients were male. Most of the patients (28/30, 93.3 %) had at least one comorbid illness. Pleural effusion was observed in 22 of the 30 patients, and pleural effusion was evaluated in 21 of 22 patients. The amount of pleural effusion in the remaining patient, who had a lung abscess, was too small to evaluate. Complicated pleural effusion and empyema were found in 10 (47.6 %) and 11 (52.4 %) patients, respectively (among the 21 patients in whom pleural effusion was examined). The numbers of patients with “pneumonia”, “lung abscess” and “bacterial pleurisy only” were 19 (63.3 %), 5 (16.7 %), 6 (20.0 %), respectively.

**Table 1:** The clinical and laboratory features of 30 patients with *Streptococcus anginosus* group infections

	Total (*n* = 30)
Age (y); mean ± SD	68.9 ± 14.2
Gender (male/female)	22/8
BMI; mean ± SD	20.1 ± 4.1
Comorbidity diseases	28 (93.3)
Neoplastic disease	8 (26.7)
Cerebrovascular disease	12 (40.0)
Chronic pulmonary disease	3 (10.0)
Chronic cardiac disease	4 (13.3)
Chronic liver disease	3 (10.0)
Chronic renal disease	4 (13.3)
Diabetes mellitus	6 (20.0)
Smoking history	13 (43.3)
Alcohol	8 (26.7)
The days from symptom onset to consultation (days)	15.1 ± 18.1
Symptoms at presentation	
Fever	24 (80.0)
Cough	14 (46.7)
Sputum	14 (46.7)
Blood sputum	2 (6.7)
Chest pain	14 (46.7)
Disturbance of consciousness	3 (10.0)
Previous antibiotic treatment	8 (26.7)
Clinical parameter	
Body temperature (°C)	37.8 ± 0.7
Systolic blood pressure (mmHg)	118.8 ± 20.9
Heart rate (beats/min)	103.7 ± 17.0
Respiratory rate (/min)	23.3 ± 5.7
Hypoxia (SpO_2_ ≤ 90 %)	18 (60.0)
Laboratory findings	
White blood cell counts (/μL)	17,627 ± 10,008
C-reactive protein (mg/dL)	18.0 ± 11.1
Albumin (g/dL)	2.5 ± 0.6
Pleural effusion	22 (73.3)
Complicated pleural effusion	10 (33.3)
Empyema	11 (36.7)
Cultured bacteria	
SAG only	24 (80.0)
Mixed infection	6 (20.0)
Pneumonia severity index	120.8 ± 42.2
Antibiotic treatment	
Monotherapy	25 (83.3)
Penicillin/beta-lactamase inhibitors	3 (10.0)
Carbapenem	19 (63.3)
Linezolid	1 (3.3)
Macrolide	2 (6.7)
Combination therapy	5 (16.7)
3th-cephem + Macrolide	1 (3.3)
4th-cephem + Clindamycin	1 (3.3)
Carbapenem + Clindamycin	2 (6.7)
Fluoroquinolone + Clindamycin	1 (3.3)
Additional treatment	
Only drainage	5 (16.7)
Drainage + lung decortication	14 (46.7)
Length of stay (days)	27.7 ± 18.8
ICU admission	6 (20.0)
In-hospital mortality	2 (6.7)

*SD* standard deviation, *BMI* body mass index, *SpO*_*2*_ pulse oximetric saturation, *SAG* *streptococcus anginosus *group, *ICU* intensive care unit

### Clinical outcomes

The antimicrobial treatment regimens and outcomes are summarized in Table 1. Antibiotic monotherapy was administered to 25 of 30 (83.3 %) patients, and 19 of 25 (63.3 %) patients were treated with carbapenems. Clindamycin was concomitantly used in combination with beta-lactam and quinolone antibiotics in 4 of the 5 (80.0 %) patients treated with combined therapy. Eleven (36.6 %) patients were treated with antibiotics only, while surgical treatments were applied (in addition to antibiotic therapy) in 19 (63.3 %) patients (Table 1). There were no significant differences in the lengths of stay or the treatment responses among the patients diagnosed with any of the three diseases. Six patients (20.0 %) were admitted to the intensive care unit (ICU) and there were two cases (6.7 %) of in-hospital mortality.

### Microbiology

*S. intermedius*, *S. constellatus*, and *S. anginosus* were detected in 16 (53.3 %), 11 (36.7 %), and 3 (10.0 %), patients, respectively (Table 2). Mixed infections with non-SAG bacteria were observed in 6 of 30 (20.0 %) patients (pneumonia; 3, lung abscess; 0, bacterial pleurisy only; 3), with mixed infections with anaerobic pathogens accounting for 3 of 30 (10.0 %) cases (Table 2).

**Table 2:** The bacteria isolated from 30 patients with *Streptococcus anginosus* group infections

Cultured bacteria	No (%)
*S. intermedius*	16 (53.3)
*S. intermedius* only	14 (46.7)
*S. intermedius* + *H. influenzae*	1 (3.3)
*S. intermedius* + *Fusobacterium* spp.	1 (3.3)
*S. constellatus*	11 (36.7)
*S. constellatus* only	8 (26.7)
*S. constellatus* + *E. coli*	1 (3.3)
*S. constellatus* + *K. pneumoniae*	1 (3.3)
*S. constellatus* + *Bacteroides* spp. + *Peptostreptococcus* spp.	1 (3.3)
*S. anginosus*	3 (10.0)
*S. anginosus* only	2 (6.7)
*S. anginosus + Bacteroides* spp.	1 (3.3)

### The clinical features, laboratory features, treatment, and outcomes among the patients with single infections

After excluding the six patients with mixed infections, 16, 5, and 3 patients were classified as having “pneumonia”, “lung abscess” and “bacterial pleurisy only”, respectively (Table 3). No significant differences were observed among three diseases with regard to age, gender, comorbid diseases and symptoms at presentation. The duration (in days) from the onset of symptoms to the patient’s hospital visit was significantly longer in the “lung abscess” patients than in the “pneumonia” patients. The “pneumonia” patients showed higher peripheral WBC counts than the “lung abscess” patients. No significant differences were observed in the numbers of detected bacteria, the treatments or the clinical outcomes of these three categories (Table 4).

**Table 3:** The clinical and laboratory features of 24 patients which was classified in pneumonia, lung abscess and bacterial pleurisy only

	Total	Pneumonia	Lung abscess	Bacterial pleurisy only	*p-*Value
	(*n* = 24)	(*n* = 16)	(*n* = 5)	(*n* = 3)	
Age (y); mean ± SD	72.1 ± 11.6	74.8 ± 9.8	68.0 ± 15.4	65.0 ± 9.8	NS
Gender (male/female)	18/6	11/5	5/0	2/1	NS
BMI; mean ± SD	20.3 ± 4.4	19.6 ± 4.6	25.0 ± 3.5	19.6 ± 2.2	*p* = 0.032^**^
Comorbidity diseases	23 (95.8)	16 (100)	4 (80.0)	3 (100)	NS
Neoplastic disease	7 (29.2)	4 (25.0)	2 (40.0)	1 (33.3)	NS
Cerebrovascular disease	9 (37.5)	7 (43.8)	1 (20.0)	1 (33.3)	NS
Chronic pulmonary disease	2 (8.3)	1 (6.3)	1 (20.0)	0 (0.0)	NS
Chronic cardiac disease	4 (16.7)	4 (25.0)	0 (0.0)	0 (0.0)	NS
Chronic liver disease	3 (12.5)	3 (18.8)	0 (0.0)	0 (0.0)	NS
Chronic renal disease	4 (16.7)	2 (12.5)	0 (0.0)	2 (66.7)	NS
Diabetes mellitus	6 (25.0)	4 (25.0)	0 (0.0)	2 (66.7)	NS
Smoking history	10 (41.7)	7 (43.8)	3 (60.0)	0 (0.0)	NS
Alcohol	6 (25.0)	5 (31.3)	1 (20.0)	0 (0.0)	NS
The days from symptom onset to consultation (days)	16.0 ± 19.6	9.0 ± 9.3	38.6 ± 30.8	16.0 ± 14.8	*p* = 0.031^*^
Symptoms at presentation					
Fever	19 (79.2)	13 (81.3)	4 (80.0)	2 (66.7)	NS
Cough	12 (50.0)	7 (43.8)	4 (80.0)	1 (33.3)	NS
Sputum	12 (50.0)	7 (43.8)	4 (80.0)	1 (33.3)	NS
Blood sputum	2 (8.3)	0 (0.0)	1 (20.0)	1 (33.3)	NS
Chest pain	12 (50.0)	9 (56.3)	1 (20.0)	2 (66.7)	NS
Disturbance of consciousness	3 (12.5)	3 (18.8)	0 (0.0)	0 (0.0)	NS
Previous antibiotic treatment	7 (29.2)	6 (37.5)	0 (0.0)	1 (33.3)	NS
Clinical parameter					
Body temperature (°C)	37.8 ± 0.80	37.9 ± 0.81	37.5 ± 0.97	37.8 ± 0.59	NS
Systolic blood pressure (mmHg)	120.0 ± 22.2	117.8 ± 22.4	116.4 ± 19.3	135.3 ± 27.2	NS
Heart rate (beats/min)	100.6 ± 15.1	102.7 ± 16.7	93.6 ± 13.4	101.0 ± 3.6	NS
Respiratory rate (/min)	23.5 ± 6.1	25.0 ± 5.5	17.3 ± 5.1	27.0 ± 4.2	*p* = 0.029^*^
Hypoxia (SpO_2_ ≤ 90 %)	15 (62.5)	12 (75.0)	1 (20.0)	2 (66.7)	*p* = 0.047^*^
Laboratory findings					
White blood cell counts (/μL)	16,763 ± 9412	18,869 ± 9532	10,080 ± 2906	16,667 ± 13,164	*p* = 0.014^*^
C-reactive protein (mg/dL)	18.2 ± 10.5	19.2 ± 11.0	11.7 ± 7.1	24.1 ± 9.6	NS
Albumin (g/dL)	2.5 ± 0.6	2.5 ± 0.7	2.7 ± 0.7	2.2 ± 0.4	NS
Pleural effusion	19 (79.2)	14 (87.5)	2 (40.0)	3 (100)	NS
Complicated pleural effusion	9 (37.5)	6 (37.5)	1 (20.0)	2 (66.7)	NS
Empyema	9 (37.5)	8 (50.0)	0 (0.0)	1 (33.3)	NS
Cultured bacteria					
*S. intermedius*	14 (58.3)	11 (68.8)	3 (60.0)	0 (0.0)	NS
*S. constellatus*	8 (33.3)	4 (25.0)	2 (40.0)	2 (66.7)	NS
*S. anginosus*	2 (8.3)	1 (6.3)	0 (0.0)	1 (33.3)	NS
Pneumonia severity index	125.2 ± 37.3	128.1 ± 37.8	123.6 ± 48.6	112.0 ± 16.5	NS

*SD* standard deviation, *BMI* body mass index, *SpO*_*2*_ pulse oximetric saturation, *NS* not significant

^*^Statistically significant difference in the comparison between pneumonia and lung abscess; ^**^Statistically significant difference in the comparison between pneumonia and bacterial pleurisy

**Table 4:** The treatments and outcomes of 24 patients which was classified in pneumonia, lung abscess and bacterial pleurisy only

	Total	Pneumonia	Lung abscess	Bacterial pleurisy only	*p-*Value
	(*n* = 24)	(*n* = 16)	(*n* = 5)	(*n* = 3)	
Monotherapy	20 (83.3)	12 (75.0)	5 (100)	3 (100)	NS
Penicillin/beta-lactamase inhibitors	1 (4.2)	1 (6.3)	0 (0.0)	0 (0.0)	NS
Carbapenem	17 (70.8)	10 (62.5)	4 (80.0)	3 (100)	NS
Linezolid	1 (4.2)	0 (0.0)	1 (20.0)	0 (0.0)	NS
Macrolide	1 (4.2)	1 (6.3)	0 (0.0)	0 (0.0)	NS
Combination therapy	4 (16.7)	4 (25.0)	0 (0.0)	0 (0.0)	NS
4th-cephem + Clindamycin	1 (4.2)	1 (6.3)	0 (0.0)	0 (0.0)	NS
Carbapenem + Clindamycin	2 (8.3)	2 (12.5)	0 (0.0)	0 (0.0)	NS
Fluoroquinolone + Clindamycin	1 (4.2)	1 (6.3)	0 (0.0)	0 (0.0)	NS
Additional treatment	16 (66.7)	12 (75.0)	1 (20.0)	3 (100)	NS
Only drainage	4 (16.7)	2 (12.5)	1 (20.0)	1 (33.3)	NS
Drainage + lung decortication	12 (50.0)	10 (62.5)	0 (0.0)	2 (66.7)	NS
Length of stay (days)	29.3 ± 19.7	31.4 ± 20.9	28.6 ± 21.0	19.0 ± 9.5	NS
ICU admission	6 (25.0)	6 (37.5)	0 (0.0)	0 (0.0)	NS
In hospital mortality	2 (8.3)	2 (12.5)	0 (0.0)	0 (0.0)	NS

*NS* not significant, *ICU* intensive care unit

### The clinical and laboratory features in patients with pleural effusion

**Fig. 2:**
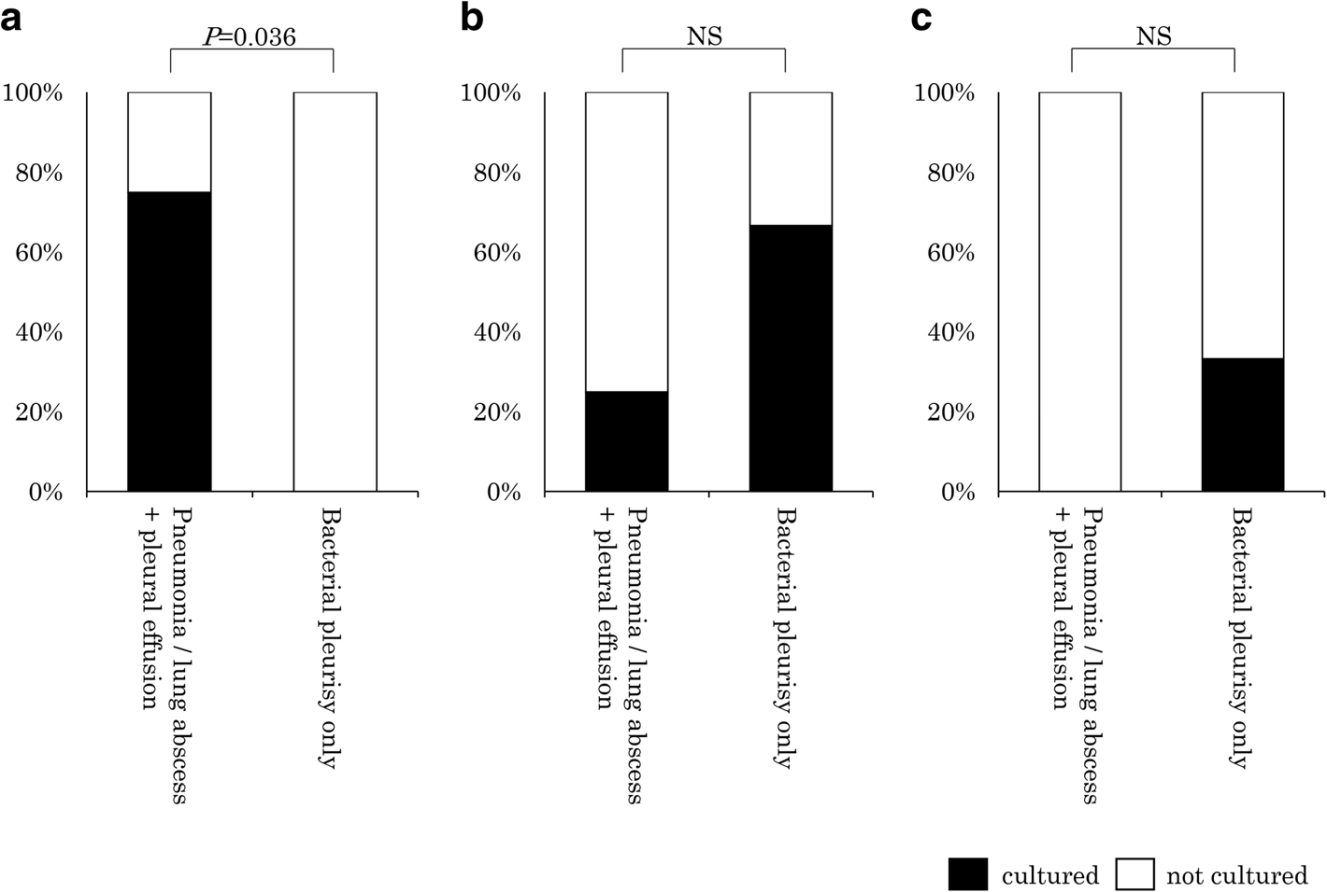
The differences in the detection rates of the three bacterial species in the pneumonia and lung abscess patients with pleural effusion (*n* = 16) and those with bacterial pleurisy only (*n* = 3). **a** *Streptococcus intermedius*, **b** *Streptococcus constellatus*, **c** *Streptococcus anginosus*

The clinical characteristics and laboratory findings of the “pneumonia” or “lung abscess” patients that were complicated with pleural effusion and the “bacterial pleurisy only” patients are shown in Additional file 1: Table S1. There were no significant differences in age, gender, comorbid diseases, symptoms at presentation, and clinical and laboratory parameters between these two categories. Whereas. *S. intermedius* was significantly more frequently identified in “pneumonia” and “lung abscess” patients with “pleural effusion” than in patients with “bacterial pleurisy only” (Fig. 2).

### The clinical and laboratory features of the different species of SAG

**Fig. 3:**
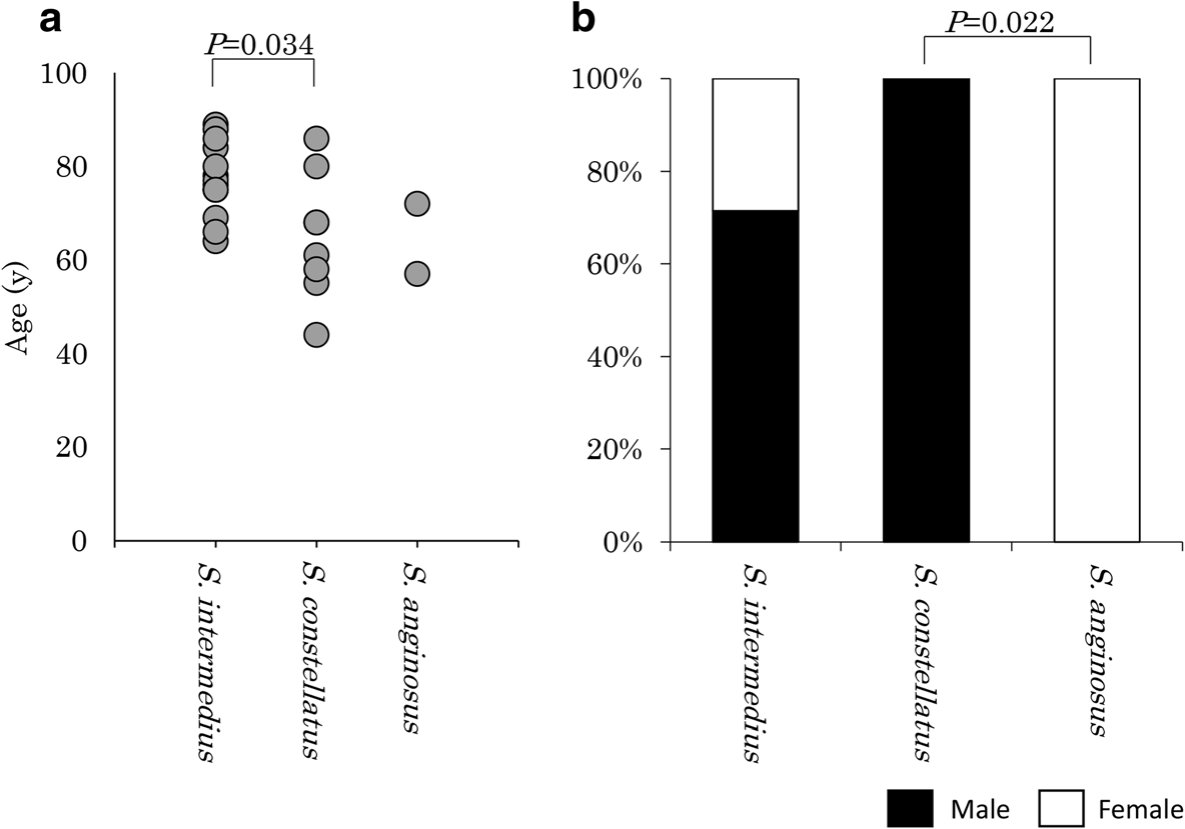
An age and gender-based comparison of each bacterial member of the *Streptococcus anginosus* group. **a** age, **b** gender

There were no significant differences in the comorbid diseases, symptoms at presentation, laboratory findings, treatments or outcomes among three SAG bacterial species (Additional file 1: Table S2). The patients with positive *S. intermedius* cultures were significantly older than those with positive *S. constellatus* cultures (Fig. 3a). All of the eight patients in whom *S. constellatus* was identified were males, and significantly more male patients were included among the patients with *S. constellatus* than those with *S. anginosus* (Fig. 3b).

## Discussion

The SAG bacteria include some of the important pathogens in respiratory infections. In the present study, we evaluated the clinical characteristics of respiratory infections caused by the SAG members using culture methods, and showed that the patients tended to be males with comorbid diseases, and that their condition tended to be complicated with pleural effusion with purulent formation. In addition, *S. intermedius* infections were mainly identified in older patients with pulmonary infections complicated with pleural effusion. Therefore, we also found that the aspiration of oral secretions may be a risk factor for the formation of empyema thoracis associated with pulmonary infection due to *S. intermedius*.

The SAG bacteria are fascultative anaerobic pathogens that ordinarily colonize the mouth and the upper respiratory tract. In this study, only 11 of 72 (15.3 %) patients were diagnosed with respiratory infections caused by SAG bacteria according to the cultivation results of lower respiratory tract specimens. The diagnosis of resiratory infections caused by SAG was made mainly based on the detection of SAG from pleural effusion, and three of these 11 patients showed the same bacterial species in both lower respiratory tract samples and pleural effusion. On the other hand, pneumonia without pleural effusion caused by SAG was only observed in 5 of 30 (16.7 %) patients with a SAG infection. This finding was in line with a similar report, which showed a low SAG detection rate in patients with pneumonia without pleural effusion [11]. In consideration of the above findings, a relatively small number of patients seems to have received a precise diagnosis of a respiratory infection caused by SAG using only lower respiratory specimens. Physicians therefore need to exercise caution, and consider their patient’s clinical background in the diagnosis of respiratory infections caused by SAG bacteria even if the SAG bacteria are cultured in samples obtained from the lower respiratory tract.

Previous reports have demonstrated that SAG infections occur mainly in male patients, patients with comorbid diseases, and pus formation, and that the mean time to pus formation is 18 days [1, 10, 20]. Similar clinical characteristics were observed in the present study. In this study, patients with pneumonia, with or without pleural effusion, tended to show a shorter period from the onset of symptoms to the diagnosis and more elevated levels of peripheral WBC counts than patients with lung abscess.

Pleural effusion was observed in 73.3 % of the patients and many of them fulfilled the diagnosis for complicated pleural effusion or empyema. Okada et al. reported that pleural effusion was observed in 54.5 % of patients with SAG infections, and that pleural effusion was more frequently observed in patients with SAG infections than in patients were infected with other pathogens, including *S. pneumoniae*, and they therefore speculated that respiratory infections caused by SAG should be especially considered in patients with pleural effusion [21]. In relation to medical treatments, early surgical treatment was necessary in 20 of 30 (66.7 %) patients. The application of early surgical treatment, in addition to appropriate antibiotic use for patients with thoracic infections caused by SAG, was associated with a low mortality rate [10], a shortened length of hospital stay and an increase in the number of patients who could be discharged to their homes [22]. The mortality rate in this study was low (6.7 %), and 19 of 30 (63.3 %) patients were treated with additional surgical treatments in combination with antibiotic treatment. Thus it should be considered that early (additional) surgical intervention might lead to favorable outcome in patients with respiratory infections caused by SAG.

Previous reports of the three different members of the SAG (*S. intermedius*, *S. constellatus* and *S. anginosus*) described that the bacteria were observed equally in thoracic infections and that there were no significant differences among these three pathogens [20, 22]. *S. intermedius* is commonly isolated from the brain or the liver, while *S. constellatus* and *S. anginosus* are widely distributed and can be found in infections of the respiratory tract or blood [2, 3]. On the other hand, higher rates of *S. intermedius* detection have also been reported from respiratory sources [23]. In addition, *S. intermedius* and *S. constellatus* are generally more frequently associated with abscess formation than *S. anginosus* [24, 25]. In this study, *S. intermedius* and *S. constellatus* were mainly detected in patients with respiratory infections. Interestingly, *S. intermedius* was significantly more common in patients with “pneumonia or lung abscess with pleural effusion” than those with “bacterial pleurisy only”, and *S. intermedius* also tended to be more frequently detected in older patients. Several mechanisms for these thoracic infections caused by SAG have been suggested, including the aspiration of oral secretions, direct implantation by trauma or surgery, extension by contiguity, and hematogenous dissemination [26]. Teramoto et al. reported that aspiration often contributes to the pathogenesis of pneumonia in elderly patients, especially those over 70–80 years of age, and an increased age is associated with the risk of developing aspiration pneumonia [27]. Therefore, the aspiration of oral secretions may be able to cause lung infection with empyema due to *S. intermedius* in older patients. In comparison with the two other SAG members, *S. constellatus* tended to be more frequently detected in male patients in this study, although there were no significant differences in the gender of patients whose cultures were positive for *S. intermedius* or *S. constellatus*. This finding is contrary to previous reports which demonstrated that there were no gender differences in the rate patients infected with these three pathogens [23]. The reasons for the gender differences between the three SAG members remain to be elucidated and further investigation is necessary.

The reported rates of mixed infections involving bacterial species other than SAG range from 13 to 45 % in patients with respiratory infections caused by the SAG bacteria [10, 12, 21, 26]. The reported rates of mixed infections involving the SAG bacteria combined with anaerobes range from 14 to 24 % [1, 5]. Anaerobes are common pathogens in mixed infections caused by the SAG bacteria; the presence of anaerobes has been reported to positively enhance SAG infection [9, 28]. In the present study, mixed infections of SAG bacteria and other bacterial species were observed in 20 % of the patients, while mixed infections of SAG bacteria and anaerobes were observed in 10 % of patients. We previously reported the clinical significance of anaerobic bacteria in bacterial pneumonia [7, 8] and pleurisy [15], and demonstrated that anaerobic pathogens may be underestimated by current culture methods [7, 8, 15]. Further studies are necessary to elucidate the clinical and pathogenic roles of the anaerobic pathogens in mixed infections involving the SAG bacteria.

The present study is associated with several limitations. First, this study was retrospective in nature and involved a relatively small sample size. Second, it was unclear whether the appropriate conditions were created for the detection of anaerobic cultures. Third, although quantitative culture methods are desirable in the bacteriological and etiological evaluation of pneumonia, we were only able to evaluate the bacterial composition semi-quantitatively.

## Conclusion

We summarized the clinical characteristics of patients with respiratory infections induced by the SAG bacteria, and found that the respiratory infections tended to more frequently occur in males with comorbid diseases and involve pleural effusion with purulent formation. *S. intermedius* was mainly identified in elderly patients with lung infection complicated with pleural effusion, and aspiration of the oral secretions may be a risk factor in the formation of empyema thoracis associated with pneumonia due to *S. intermedius*. In addition to investigating culture results, physicians should pay more attention to the clinical background, including symptoms such as pleural effusion.

## Additional file

Additional file 1: Table S1. The clinical and laboratory features of patients with pneumonia/lung abscess with pleural effusion and bacterial pleurisy only. **Table S2.** The clinical and laboratory features of patients with *Streptococcus anginosus* group infections. (DOCX 28 kb)

## Abbreviations

SAG: *Streptococcus anginosus *group
WBC: White blood cell count
CRP: C-reactive protein
BALF: Bronchoalveolar lavage fluid
