# Supplementary material for: The clinical features of respiratory infections caused by the Streptococcus anginosus group
Source: BMC Pulm Med. 2015 Oct 26;15:133. doi: 10.1186/s12890-015-0128-6 (PMC4624190; doi:10.1186/s12890-015-0128-6)
Supplement: Additional file 1: Table S1. — The clinical and laboratory features of patients with pneumonia/lung abscess with pleural effusion and bacterial pleurisy only. Table S2. The clinical and laboratory features of patients with Streptococcus anginosus group infections. (DOCX 28 kb) [file 12890_2015_128_MOESM1_ESM.docx]

Additional file 1

Table S1. The clinical and laboratory features of patients with pneumonia/lung abscess with pleural effusion and bacterial pleurisy only

|  |  | Pneumonia / lung abscess ＋pleural effusion | | Bacterial pleurisy only | | *p-*Value |
| --- | --- | --- | --- | --- | --- | --- |
|  |  | (n=16) | | (n=3) | |  |
| Age (y); mean ± SD | | 74.3±10.0 | | 65.0±13.0 | | NS |
| Gender (male / female) | | 13 / 3 | | 2 / 1 | | NS |
| BMI; mean ± SD | | 18.9±2.9 | | 25.0±3.5 | | *p* =0.017 |
| Comorbidity diseases | | 15 | (93.8) | 3 | (100) | NS |
|  | Neoplastic disease | 5 | (31.3) | 1 | (33.3) | NS |
|  | Cerebrovascular disease | 6 | (37.5) | 1 | (33.3) | NS |
|  | Chronic pulmonary disease | 0 | (0.0) | 0 | (0.0) | NS |
|  | Chronic cardiac disease | 4 | (25.0) | 0 | (0.0) | NS |
|  | Chronic liver disease | 3 | (18.8) | 0 | (0.0) | NS |
|  | Chronic renal disease | 2 | (12.5) | 2 | (66.7) | NS |
|  | Diabetes mellitus | 3 | (18.8) | 2 | (66.7) | NS |
| Smoking history | | 8 | (50.0) | 0 | (0.0) | NS |
| Alcohol | | 5 | (31.3) | 0 | (0.0) | NS |
| The days from symptom onset to consultation (days) | | 15.9 ± 21.9 | | 16.0 ±14.8 | | NS |
| Symptoms at presentation | |  |  |  |  |  |
|  | Fever | 12 | (75.0) | 2 | (66.7) | NS |
|  | Cough | 8 | (50.0) | 1 | (33.3) | NS |
|  | Sputum | 7 | (43.8) | 1 | (33.3) | NS |
|  | Blood sputum | 0 | (0.0) | 1 | (33.3) | NS |
|  | Chest pain | 9 | (56.3) | 2 | (66.7) | NS |
|  | Disturbance of consciousness | 2 | (12.5) | 0 | (0.0) | NS |
| Previous antibiotic treatment | | 5 | (31.3) | 1 | (33.3) | NS |
| Clinical parameter | |  |  |  |  |  |
|  | Body temperature (°C) | 37.8±0.84 | | 37.8±0.6 | | NS |
|  | Systolic blood pressure (mmHg) | 121.2±22.3 | | 135.3±27.2 | | NS |
|  | Hear rate (beats/min) | 103.7±16.4 | | 101.0±3.6 | | NS |
|  | Respiratory rate (/min) | 25.2±5.7 | | 27.0±4.2 | | NS |
|  | Hypoxia (SpO_2_ ≤ 90%) | 12 | (75.0) | 2 | (66.7) | NS |
| Laboratory findings | |  |  |  |  |  |
|  | White blood cell counts (/μL) | 19,156±9,159 | | 16,666±13,164 | | NS |
|  | C-reactive protein (mg/dL) | 19.2±11.1 | | 24.1±9.6 | | NS |
|  | Albumin (g/dL) | 2.4±0.63 | | 2.2±0.4 | | NS |
| Cultured bacteria | |  |  |  |  |  |
|  | *S. intermedius* | 12 | (75.0) | 0 | (0.0) | *p* =0.036 |
|  | *S.constellatus* | 4 | (25.0) | 2 | (66.7) | NS |
|  | *S. anginosus* | 0 | (0.0) | 1 | (33.3) | NS |
| Pleural effusion | |  |  |  |  |  |
|  | Complicated pleural effusion | 7 | (43.8) | 2 | (66.7) | NS |
|  | Empyema | 8 | (50.0) | 1 | (33.3) | NS |
| Length of stay (days) | | 31.4±20.9 | | 19.0±9.5 | | NS |
| ICU admission | | 6 | (37.5) | 0 | (0.0) | NS |
| In hospital mortality | | 2 | (12.5) | 0 | (0.0) | NS |
| Abbreviations: SD, standard deviation; BMI, body mass index; SpO_2_, pulse oximetric saturation; NS, not significant; ICU, intensive care unit | | | | | | |

Table S2. The clinical and laboratory features of patients with *Streptococcus anginosus* group infections

|  |  | *S.intermedius* | | *S.constellatus* | | *S.anginosus* | | *p-*Value |
| --- | --- | --- | --- | --- | --- | --- | --- | --- |
|  |  | (n=14) | | (n=8) | | (n=2) | |  |
| Age (y); mean ± SD | | 77.3±7.7 | | 65.0±13.6 | | 64.5±10.6 | | *p* = 0.034^§1^ |
| Gender (male / female) | | 10 / 4 | | 8 / 0 | | 0 / 2 | | *p* = 0.022^§2^ |
| BMI; mean ± SD | | 19.3±2.4 | | 19.2±3.4 | | 30.9±2.8 | | *p* = 0.014^§3^ |
| Comorbidity diseases | | 13 | (92.9) | 8 | (100) | 2 | (100) | NS |
|  | Neoplastic disease | 4 | (28.6) | 2 | (25.0) | 1 | (50.0) | NS |
|  | Cerebrovascular disease | 4 | (28.6) | 5 | (62.5) | 0 | (0.0) | NS |
|  | Chronic pulmonary disease | 1 | (7.1) | 1 | (12.5) | 0 | (0.0) | NS |
|  | Chronic cardiac disease | 4 | (28.6) | 0 | (0.0) | 0 | (0.0) | NS |
|  | Chronic liver disease | 2 | (14.3) | 1 | (12.5) | 0 | (0.0) | NS |
|  | Chronic renal disease | 2 | (14.3) | 2 | (25.0) | 0 | (0.0) | NS |
|  | Diabetes mellitus | 2 | (14.3) | 3 | (37.5) | 1 | (50.0) | NS |
| Smoking history | | 7 | (50.0) | 3 | (37.5) | 0 | (0.0) | NS |
| Alcohol | | 5 | (35.7) | 1 | (12.5) | 0 | (0.0) | NS |
| The days from symptom onset to consultation (days) | | 18.9±23.1 | | 10.4±12.7 | | 18.5±20.5 | | NS |
| Symptoms at presentation | |  |  |  |  |  |  |  |
|  | Fever | 10 | (71.4) | 7 | (87.5) | 2 | (100) | NS |
|  | Cough | 8 | (57.1) | 2 | (25.0) | 2 | (100) | NS |
|  | Sputum | 7 | (50.0) | 3 | (37.5) | 2 | (100) | NS |
|  | Blood sputum | 0 | (0.0) | 1 | (12.5) | 1 | (50.0) | NS |
|  | Chest pain | 6 | (42.9) | 6 | (75.0) | 0 | (0.0) | NS |
|  | Disturbance of consciousness | 3 | (21.4) | 0 | (0.0) | 0 | (0.0) | NS |
| Previous antibiotic treatment | | 5 | (35.7) | 1 | (12.5) | 1 | (50.0) | NS |
| Clinical parameter | |  |  |  |  |  |  |  |
|  | Body temperature (°C) | 37.7±0.8 | | 37.9±0.9 | | 38.2±0.1 | | NS |
|  | Systolic blood pressure (mmHg) | 117.5±22.6 | | 125.4±24.5 | | 112.0±2.8 | | NS |
|  | Hear rate (beats/min) | 102.0±15.7 | | 99.8±16.1 | | 94.0±8.5 | | NS |
|  | Respiratory rate (/min) | 25.3±6.5 | | 19.7±4.1 | | 26.0±5.7 | | NS |
|  | Hypoxia (SpO_2_ ≤ 90%) | 11 | (78.6) | 3 | (37.5) | 1 | (50.0) | NS |
| Laboratory findings | |  |  |  |  |  |  |  |
|  | White blood cell counts (/μL) | 18,071±10,112 | | 14,300±5,715 | | 17,450±20,152 | | NS |
|  | C-reactive protein (mg/dL) | 17.6±10.6 | | 21.9±10.0 | | 8.0±8.1 | | NS |
|  | Albumin (g/dL) | 2.4±0.6 | | 2.6±0.5 | | 2.7±1.3 | | NS |
| Radiographic findings | |  |  |  |  |  |  |  |
|  | Pleural effusion | 12 | (85.7) | 6 | (75.0) | 1 | (50.0) | NS |
| Pneumonia | | 11 | (78.6) | 4 | (50.0) | 1 | (50.0) | NS |
| Lung abscess | | 3 | (21.4) | 2 | (25.0) | 0 | (0.0) | NS |
| Bacterial pleurisy only | | 0 | (0.0) | 2 | (25.0) | 1 | (50.0) | NS |
| Antibiotic treatment | |  |  |  |  |  |  |  |
| Monotherapy | | 10 | (71.4) | 8 | (100) | 2 | (100) | NS |
|  | Penicillin/beta-lactamase inhibitors | 1 | (7.1) | 0 | (0.0) | 0 | (0.0) | NS |
|  | Carbapenem | 9 | (64.3) | 7 | (87.5) | 1 | (50.0) | NS |
|  | Linezolid | 0 | (0.0) | 1 | (12.5) | 0 | (0.0) | NS |
|  | Macrolide | 0 | (0.0) | 0 | (0.0) | 1 | (50.0) | NS |
| Combination therapy | | 4 | (28.6) | 0 | (0.0) | 0 | (0.0) | NS |
|  | 3th-cephem + macrolide | 0 | (0.0) | 0 | (0.0) | 0 | (0.0) | NS |
|  | 4th-cephem + clindamycin | 1 | (7.1) | 0 | (0.0) | 0 | (0.0) | NS |
|  | Carbapenem + clindamycin | 2 | (14.3) | 0 | (0.0) | 0 | (0.0) | NS |
|  | Fluoroquinolone + clindamycin | 1 | (7.1) | 0 | (0.0) | 0 | (0.0) | NS |
| Additional treatment | | 9 | (64.3) | 6 | (75.0) | 1 | (50.0) | NS |
|  | Only drainage | 2 | (14.3) | 1 | (12.5) | 1 | (50.0) | NS |
|  | Drainage + lung decortication | 7 | (50.0) | 5 | (62.5) | 0 | (0.0) | NS |
| Length of stay (days) | | 32.5±21.8 | | 26.6±17.1 | | 17.5±14.8 | | NS |
| ICU admission | | 5 | (35.7) | 1 | (12.5) | 0 | (0.0) | NS |
| In-hospital mortality | | 2 | (14.3) | 0 | (0.0) | 0 | (0.0) | NS |
| Abbreviations: SD, standard deviation; BMI, body mass index; SpO_2_, pulse oximetric saturation; NS, not significant; ICU, intensive care unit  ^§1^ Statistically significant difference in the comparison between *S. intermedius* and *S. constellatus*  ^§2^ Statistically significant difference in the comparison between *S. constellatus* and *S. anginosus*  ^§3^ Statistically significant difference in the comparison between *S. anginosus* and *S. intermedius* | | | | | | | | |
